# Prevalence of right ventricular dysfunction and prognostic significance in heart failure with preserved ejection fraction

**DOI:** 10.1007/s10554-020-01953-y

**Published:** 2020-07-31

**Authors:** Prathap Kanagala, Jayanth R. Arnold, Anvesha Singh, Jamal N. Khan, Gaurav S. Gulsin, Pankaj Gupta, Iain B. Squire, Leong L. Ng, Gerry P. McCann

**Affiliations:** 1 Department of Cardiovascular Sciences, National Institute for Health Research (NIHR) Leicester Biomedical Research Centre, University of Leicester, Leicester, UK; 2 Aintree University Hospital, Liverpool, UK; 3 Department of Cardiovascular Sciences, Glenfield Hospital, Groby Road, Leicester, LE3 9QP UK

**Keywords:** Heart failure with preserved ejection fraction, Right ventricular dysfunction, Prevalence, Prognosis, Cardiovascular magnetic resonance imaging

## Abstract

There is a paucity of data characterizing right ventricular performance in heart failure with preserved ejection fraction (HFpEF) using the gold standard of cardiovascular magnetic resonance imaging (CMR). We aimed to assess the proportion of right ventricular systolic dysfunction (RVD) in HFpEF and the relation to clinical outcomes. As part of a single-centre, prospective, observational study, 183 subjects (135 HFpEF, and 48 age- and sex-matched controls) underwent extensive characterization with CMR. transthoracic echocardiography, blood sampling and six-minute walk testing. Patients were followed for the composite endpoint of death or HF hospitalization. RVD (defined as right ventricular ejection fraction < 47%) controls was present in 19% of HFpEF. Patients with RVD presented more frequently with lower systolic blood pressure, atrial fibrillation, radiographic evidence of pulmonary congestion and raised cardiothoracic ratio and larger right ventricular volumes. During median follow-up of 1429 days, 47% (n = 64) of HFpEF subjects experienced the composite endpoint of death (n = 22) or HF hospitalization (n = 42). RVD was associated with an increased risk of composite events (Log-Rank p = 0.001). In multivariable Cox regression analysis, RVD was an independent predictor of adverse outcomes (adjusted Hazard Ratio [HR] 3.946, 95% CI 1.878–8.290, p = 0.0001) along with indexed extracellular volume (HR 1.742, CI 1.176–2.579, p = 0.006) and E/E’ (HR 1.745, CI 1.230–2.477, p = 0.002). RVD as assessed by CMR is prevalent in nearly one-fifth of HFpEF patients and is independently associated with death and/or hospitalization with HF.

The trial was registered retrospectively on ClinicalTrials.gov (Identifier: NCT03050593). The date of registration was February 06, 2017.

**Electronic supplementary material:**

The online version of this article (10.1007/s10554-020-01953-y) contains supplementary material, which is available to authorized users.

## Introduction

The importance of right ventricular (RV) function and its impact upon functional status [1] and outcomes [2] in heart failure with reduced ejection fraction (HFrEF) is well established. However, heart failure with preserved ejection fraction (HFpEF) currently accounts for approximately half of all cases of heart failure [3] and the role of right ventricular systolic dysfunction (RVD) in this setting is less well studied. To date, the majority of evidence for RVD is largely derived from echocardiographic data [4]. Moreover, the reported prevalence of RVD in HFpEF varies depending upon the choice of RV assessment tool and differing diagnostic thresholds (e.g. tricuspid annular plane systolic excursion [TAPSE], fractional area change [FAC], right ventricular ejection fraction [RVEF]) [5].

Cardiovascular magnetic resonance imaging (CMR) is the gold standard for RV volumetric and functional assessment, providing excellent accuracy and reproducibility [6, 7]. However, only 2 CMR studies [8, 9] have assessed RV function in in HFpEF, again with differing thresholds for RVD and both lacking reference control groups. All of the above observations were recognized in a position statement from the Heart Failure Association of the European Society of Cardiology, proposing further prospective outcome studies to identify clear cut-off values for RVD that are prognostically and clinically relevant [4]. In our prospective, observational study comprising both groups of HFpEF and age- and sex-matched healthy subjects, we aimed to assess the proportion of patients with RVD and explored the relation to clinical outcomes.

## Materials and methods

### Study population

HFpEF patients were recruited as part of the Developing Imaging And plasMa biOmarkers iN Describing HFpEF (DIAMOND-HFpEF) study: an observational, cohort study conducted at a single tertiary cardiac centre [10]. The National Research Ethics Service approved the study. All subjects provided written informed consent prior to participation. As detailed previously [11, 12] HFpEF inclusion criteria were: clinical or radiographic evidence of heart failure (HF), left ventricular (LV) ejection fraction (EF) > 50% on transthoracic echocardiography (TTE) and age ≥ 18 years. Exclusion criteria were: known myocardial infarction (MI) in the preceding 6 months; suspected or confirmed cardiomyopathy or constrictive pericarditis; non-cardiovascular life expectancy < 6 months; severe native valve disease; severe lung disease (or forced expiratory volume [FEV_1_] < 30% predicted or forced vital capacity [FVC] < 50% predicted); estimated glomerular filtration rate (eGFR) < 30 ml/min/m^2^ and standard contraindications to CMR.

A control group of 48 asymptomatic, age- and sex-matched subjects without known cardiac disease were also recruited. Since hypertension is highly prevalent in the general population without heart failure and is also strongly associated with incident HFpEF development, we included a subset of hypertensive controls (n = 22) in order to account for this potential confounder. All study participants underwent comprehensive clinical evaluation, blood sampling, TTE, six-minute walk testing (6MWT) and CMR during a solitary study visit.

### Blood sampling

Blood was sampled for measurement of B-type natriuretic peptide (BNP; immunoassay, Siemens, Erlangen, Germany), haematocrit, haemoglobin and renal function (urea, creatinine).

### Transthoracic echocardiography

As reported previously [11, 12] image acquisition and analysis was undertaken by 2 experienced, accredited sonographers using an iE 33 system with S5-1 transducer (Philips Medical Systems, Best, The Netherlands). Echocardiography was performed primarily to confirm preserved LVEF for study inclusion and E/E′ was also calculated to assess LV filling pressure.

### Functional assessment of exercise capacity

To provide an objective metric of exercise capacity, the 6MWT distance was measured in all subjects according to standardized protocols [13].

### Chest radiography

The radiology reports of the most recent chest X-ray prior to the study visits were sourced from the hospital computerized reporting system [10]. The presence of pulmonary congestion and an enlarged cardiothoracic ratio were recorded. All reporting was done by Radiologists blinded to study participation and prior to subject enrolment.

### CMR protocol

The CMR protocol used has been reported previously [11, 14, 15]. All scans were performed on a 3 T scanner (Siemens Skyra, Erlangen, Germany) with an 18-channel cardiac coil. In summary, the protocol included: conventional long- and short-axis cine images covering the LV and RV; pre- and post-contrast T1 mapping of basal, mid and apical LV slices; and late gadolinium enhancement (LGE) imaging. The total contrast dose administered was 0.15 mmol/kg of Gadovist (Bayer Healthcare, Berlin, Germany).

### CMR image analysis

**Fig. 1:**
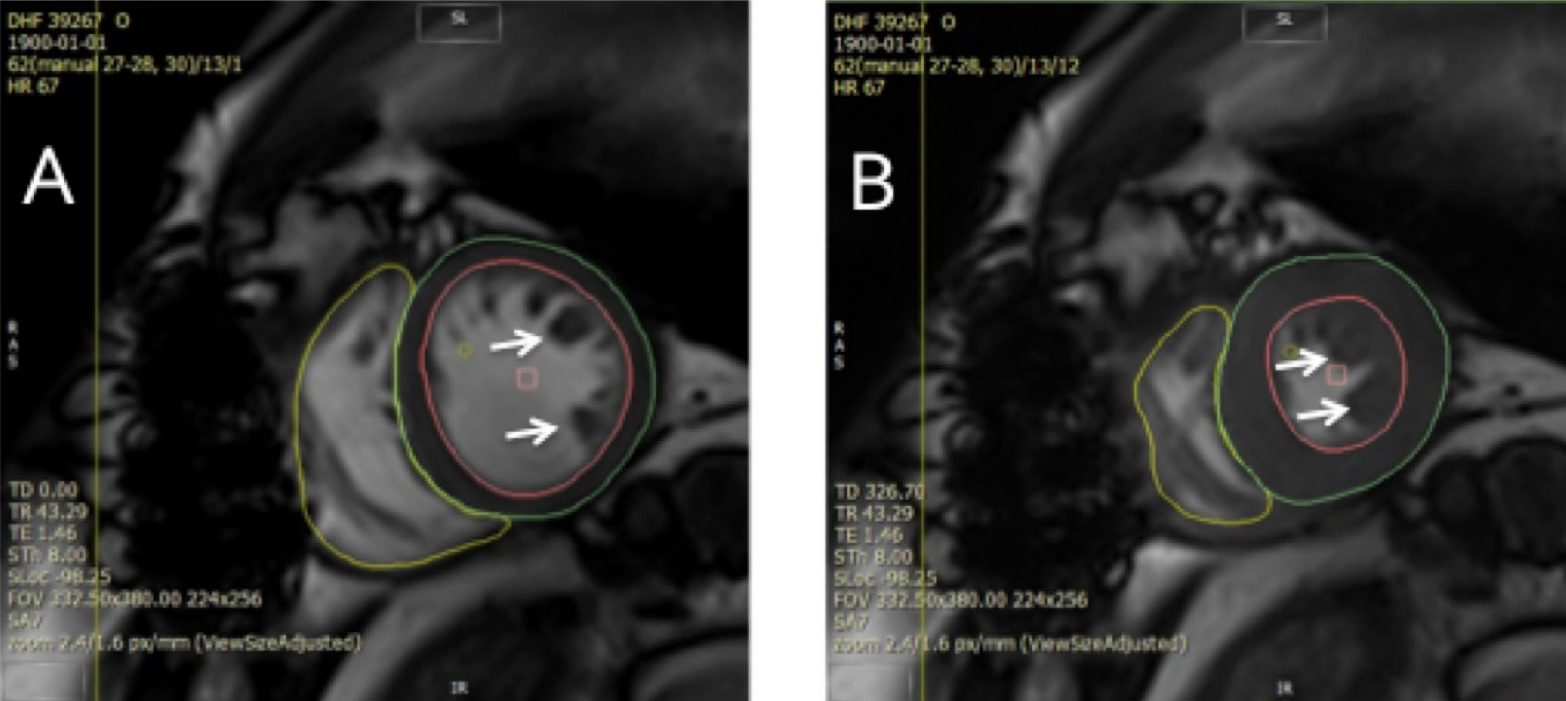
Assessment of ventricular volumes, function and mass. End-diastolic (**a**) and end-systolic (**b**) cine frames illustrating manually drawn contours of the left ventricular endocardium (pink), left ventricular epicardium (green) and right ventricular endocardium (yellow) for volumetric and mass analysis. (white arrows) papillary muscles and trabeculations were excluded from left ventricular mass calculations

Cine images were analyzed using semi-automated *cvi42* software (Circle Cardiovascular Imaging, Calgary, Canada) by a single experienced observer (PK), blinded to all clinical data. All volumetric and mass data were indexed to body surface area (BSA). Ventricular volumes (see Fig. 1), ejection fraction and LV mass (excluding papillary muscles) were calculated from the short-axis cine stack as previously described [10, 15]. The biplane method, excluding the appendage and pulmonary veins was used to measure maximal, minimal left atrial volumes (LAVmax, LAVmin) and derive left atrial ejection fraction (LAEF) [11]. A cut-off RVEF value of < 47% was used to define RVD based upon existing normative data from the published literature utilizing the same technique as in our study [16] as well as from our own healthy control data whereby the lower limit of RVEF was also 47%. Qualitative LGE assessment was undertaken by 2 experienced operators to define the presence of MI as per standard criteria [15, 17]. In cases of disagreement, final adjudication was deferred to a third operator (GPM). As previously reported by our group with excellent reproducibility [18] and intra/inter-observer agreements [15], extracellular volume (ECV) and indexed ECV were quantified from mid short-axis LV slice T1 maps.

### Outcome data

All participants were followed up for the primary endpoint, a composite of all-cause mortality or HF hospitalization (defined as a hospital admission for HF which required diuretic, inotropic or intravenous nitrate therapy). In patients experiencing multiple events, the time to first event was used as the censored outcome. Outcome data were obtained from Hospital databases. All patients had a minimum of 12 months follow-up, post study entry.

### Statistical analysis

Statistical analyses were performed using SPSS V22. A p value of < 0.05 was considered significant. Normality for continuous data was assessed using histograms, Q-Q plots and the Shapiro–Wilk test. Continuous data are presented as mean (± standard deviation) or median (25–75% interquartile range or range). Categorical data are presented as absolute numbers or percentages. Between group differences were compared using the *t* test, Mann–Whitney U test and the Chi-square test as appropriate. BNP, creatinine, 6MWT distance and RVEF were log_10_ transformed before analysis.

Spearman’s rank correlations were performed to check for important associations of other continuous variables with RVEF. Intra-observer and inter-observer variability assessments for RV parameters were undertaken (by PK and JRA), a minimum of 4 weeks apart on 10 randomly selected patients.

Event rates were calculated from Kaplan–Meier survival analysis. Survival curve differences were assessed using the Log-Rank test. Univariable Cox regression modeling was initially performed to identify variables associated with outcomes. Only parameters associated with endpoints at p < 0.1 were entered into subsequent multivariable analysis to identify independent predictors using both backwards and forwards stepwise elimination methods. In cases of collinearity, only variables with the highest coefficients or which have shown historically stronger prognostic importance based upon published literature were entered into multivariable analysis. To further minimize over-fitting, Cox regression models were limited to one parameter per approximately 10 composite events. Four separate, clinically relevant models were created including a final model comprising the strongest predictors. Continuous variables were further Z-standardized to enable comparison of hazard ratios (HR) based upon one standard deviation increase in the predictor variable. The accuracy of the final independent Cox model to predict events was then tested by receiver operator characteristics (ROC) analysis.

## Results

**Fig. 2:**
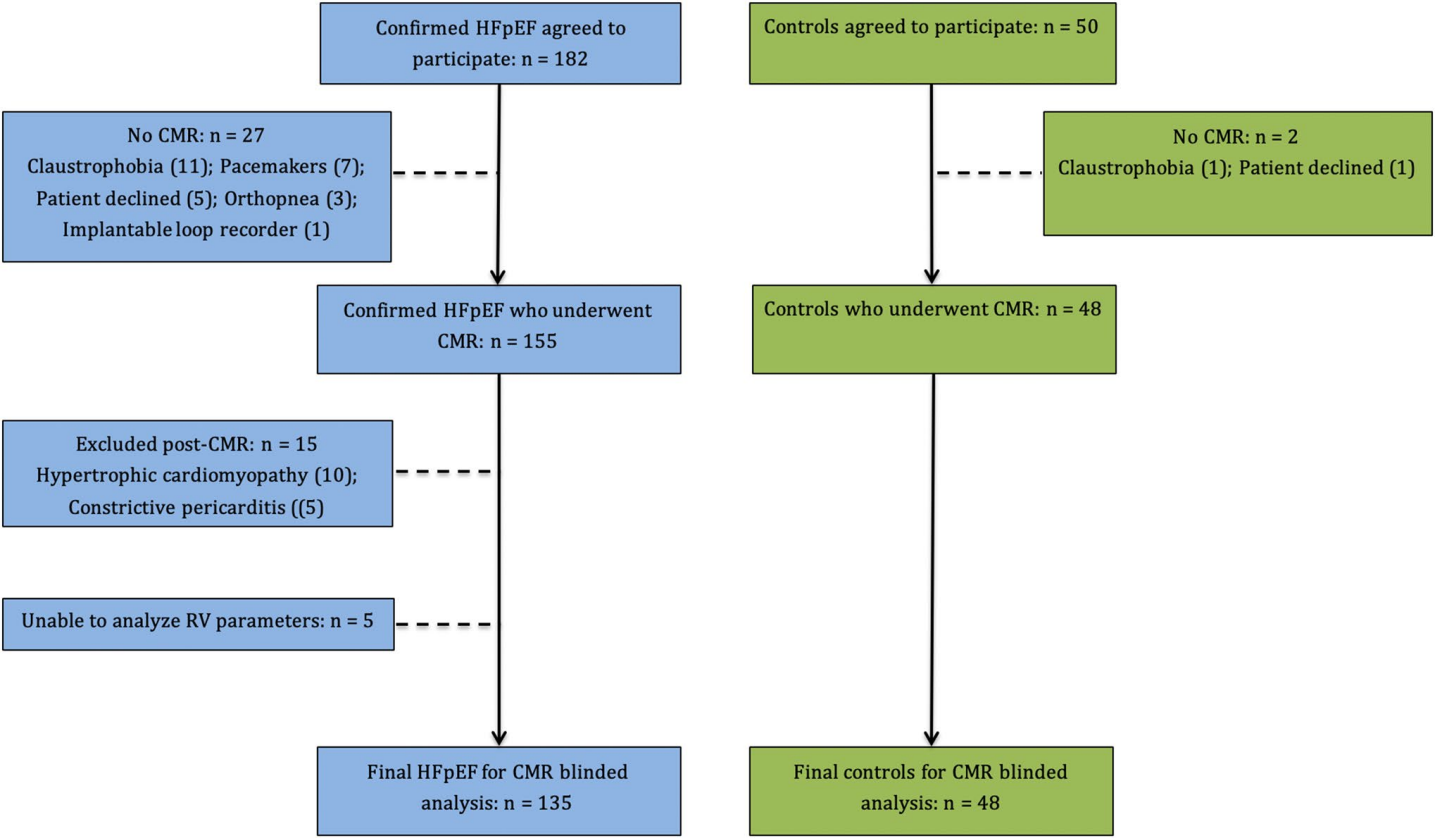
Study recruitment overview. Flow chart illustrating recruitment and reasons for exclusion. *CMR* cardiovascular magnetic resonance imaging, *HFpEF* heart failure with preserved ejection fraction, *RV* right ventricle

Two hundred and thirty two subjects were enrolled (HFpEF n = 182, controls n = 50), of whom 49 were excluded (see Fig. 2) from further analysis. Of these, RV assessment could not be performed in 5 patients due to degraded image quality. Our final cohort who underwent RV analysis comprised a total of 183 participants (HFpEF n = 135, controls n = 48). As previously reported, iECV calculation was not possible (HFpEF n = 43, controls n = 4) in a small subset due to the unavailability of the sequences for T1 mapping [15]. Both intra-observer and inter-observer variability were excellent for RV parameters [10]. All subjects were recruited over a period of 26 months. The final participant was enrolled in April 2015. Follow-up was until January 2019. Baseline demographic features of patients with HFpEF and control subjects are shown in Table1; imaging data are in Table 2.

**Table 1:** Baseline clinical characteristics

	Controls n = 48	HFpEF n = 135	p value	HFpEF No RVD n = 110	HFpEF with RVD n = 25	p value
Demographics						
Age (years)	73 ± 5	72 ± 9	0.521	72 ± 9	75 ± 11	0.183
Male (%)	24 (50)	66 (49)	0.895	51 (46)	15 (60)	0.218
Clinical findings						
Heart rate (b.p.m.)	68 ± 10	70 ± 14	0.195	70 ± 14	70 ± 14	0.991
Systolic BP (mmHg)	151 ± 24	145 ± 25	0.193	147 ± 25	136 ± 26	0.042
Diastolic BP (mmHg)	79 ± 10	74 ± 12	0.016	74 ± 12	74 ± 14	0.924
Body mass index (kg/m^2^)	25 ± 3	34 ± 7	< 0.0001	34 ± 7	33 ± 7	0.623
Sinus rhythm (%)	48 (100)	94 (70)	< 0.0001	82 (75)	12 (48)	0.009
Atrial fibrillation (%)	0 (0)	41 (30)	< 0.0001	28 (25)	13 (52)	0.009
Medical history						
Prior hospitalization with heart failure	NA	89 (66)	NA	67 (61)	22 (88)	0.010
Diabetes (%)	0 (0)	67 (50)	< 0.0001	54 (49)	13 (52)	0.793
Hypertension (%)	22 (46)	122 (90)	< 0.0001	97 (88)	25 (100)	0.071
Angina (%)	0 (0)	22 (16)	0.003	18 (16)	4 (16)	0.965
Known myocardial infarction (%)	0 (0)	15 (11)	0.016	13 (12)	2 (8)	0.583
Known coronary artery disease (%)	0 (0)	30 (22)	< 0.0001	24 (22)	6 (24)	0.813
Asthma or COPD (%)	3 (6)	21 (16)	0.101	16 (15)	5 (20)	0.497
Smoking (%)	17 (35)	71 (53)	0.041	59 (54)	12 (48)	0.610
Hypercholesterolameia (%)	18 (38)	68 (50)	0.125	56 (51)	12 (48)	0.793
Peripheral vascular disease (%)	0 (0)	3 (2)	0.298	2 (2)	1 (4)	0.504
TIA or CVA (%)	1 (2)	19 (14)	0.005	16 (15)	3 (1)	0.770
Medication						
Betablocker (%)	2 (4)	93 (69)	< 0.0001	72 (65)	21 (84)	0.071
ACEi or ARB (%)	10 (21)	116 (86)	< 0.0001	95 (86)	21 (84)	0.759
Aldosterone antagonist (%)	0 (0)	42 (31)	< 0.0001	32 (29)	10 (40)	0.288
Loop diuretic (%)	0 (0)	108 (80)	< 0.0001	86 (78)	22 (88)	0.268
Functional status						
NYHA I/II (%)	NA	95 (70)	NA	80 (75)	15 (60)	0.208
NYHA III/IV (%)	NA	40 (30)	NA	30 (27)	10 (40)	0.208
Six minute walk distance (m)	380 (350–440)	185 (120–250)	< 0.0001	190 (130–250)	180 (100–260)	0.633
Bloods						
Sodium (mmol/l)	140.4 ± 1.7	139.3 ± 3.5	0.007	139.2 ± 3.3	139.6 ± 4.2	0.661
Urea (mmol/l)	6.1 ± 1.5	8.3 ± 3.4	< 0.0001	8.4 ± 3.4	8.0 ± 3.5	0.613
Creatinine (mmol/l, IQR)	70.5 (56.3–84.5)	88 (73–113)	< 0.0001	90 (73–116)	84 (70–108)	0.283
Haemoglobin (g/l)	140 ± 15	129 ± 22	< 0.0001	129 ± 22	127 ± 21	0.658
BNP (ng/l, IQR)	33 (24–44)	136 (65–256)	< 0.0001	134 (54–269)	170 (84–245)	0.428

Values are mean ± SD or n (%) or median (interquartile range). The p values are for the t-test, Mann–Whitney U test or Chi-square test as appropriate

*ACEi* angiotensin converting enzyme inhibitor, *ARB* angiotensin II receptor blocker, *BNP* B-type natriuretic peptide, *CMR* cardiovascular magnetic resonance imaging, *COPD* chronic obstructive pulmonary disease, *CVA* cerebrovascular accident, *NA* not applicable or not assessed, *NYHA* New York Heart association, *TIA* transient ischaemic attack

**Table 2:** Baseline imaging characteristics

	Controls n = 48	HFpEF n = 135	p value	HFpEF No RVD n = 110	HFpEF with RVD n = 25	p value
Previous chest radiography						
Pulmonary congestion (%)	NA	93 (69)	NA	71 (65)	22 (88)	0.025
Raised CTR (%)	NA	98 (73)	NA	75 (68)	23 (92)	0.018
Pleural effusion (%)	NA	48 (36)	NA	36 (33)	12 (48)	0.159
Echo						
E/E′	9 ± 3	13 ± 5	< 0.0001	13 ± 5	13 ± 6	0.723
CMR						
LVEF (%)	58 ± 5	56 ± 5	0.019	56 ± 5	55 ± 6	0.449
LVEDVI (ml/m^2^)	81 ± 14	79 ± 18	0.409	79 ± 19	77 ± 16	0.493
LVMI (g/m^2^)	46 ± 9	52 ± 15	< 0.0001	52 ± 16	52 ± 10	0.886
LV mass/LV volume	0.57 ± 0.09	0.68 ± 0.16	< 0.0001	0.67 ± 0.16	0.70 ± 0.15	0.447
RVEF (%), median, range	55 (47–70)	54 (27–74)	0.090	56 (47–74)	44 (27–46)	< 0.0001
RVEDVI (ml/m^2^)	83 ± 15	80 ± 19	0.307	76 ± 16	98 ± 20	< 0.0001
RVESVI (ml/m^2^)	37 ± 9	37 ± 14	0.849	33 ± 10	57 ± 15	< 0.0001
Overall						
LAEF (%)	51 ± 11	32 ± 16	< 0.0001	35 ± 15	22 ± 12	< 0.0001
LAVImax (ml/m^2^)	35 ± 12	53 ± 25	< 0.0001	51 ± 23	62 ± 31	0.054
LAVImin (ml/m^2^)	17 ± 8	38 ± 25	< 0.0001	36 ± 23	49 ± 31	0.017
Sinus rhythm						
LAEF (%)	51 ± 11	41 ± 12	< 0.0001	42 ± 11	32 ± 10	0.006
LAVImax (ml/m^2^)	35 ± 12	43 ± 16	0.003	43 ± 16	44 ± 17	0.854
LAVImin (ml/m^2^)	17 ± 8	26 ± 13	< 0.0001	25 ± 13	30 ± 15	0.243
Presence of MI on LGE (%)	0 (0)	23 (17)	0.002	17 (15)	6 (24)	0.305
iECV (ml/m^2^)	10.9 ± 2.8	13.7 ± 4.4	< 0.0001	13.5 ± 4.5	14.7 ± 3.7	0.276

Values are mean ± SD or n (%). The p values are for the t-test, Mann–Whitney U test or Chi-square test as appropriate

*CTR* cardiothoracic ratio, *iECV* indexed extracellular volume, *LAEF* left atrial ejection fraction, *LAVI* left atrial volume indexed to body surface area (maximal/minimal), *LGE* late gadolinium enhancement, *LVMI* left ventricular end-diastolic mass indexed to body surface area, *LVEDVI* left ventricular end-diastolic volume indexed to body surface area, *MI* myocardial infarction, *NA* not applicable or not assessed, *RVEF* right ventricular ejection fraction, *RVEDVI* right ventricular end-diastolic volume indexed to body surface area, *RVESVI* right ventricular end-systolic volume indexed to body surface area

### Comparison of HFpEF and controls

Overall, HFpEF and healthy controls were well matched for age (73 ± 9 years) and sex. Approximately two-thirds of HFpEF patients had experienced prior hospitalization for HF or had radiographic evidence of pulmonary congestion. HFpEF was frequently associated with co-morbidities including obesity, diabetes, hypertension, atrial fibrillation (AF), renal dysfunction and anaemia. Significant minorities of HFpEF also had known ischaemic heart disease (22%) and lung disease (16%). Furthermore, HFpEF patients had worse exercise capacity (shorter 6MWT distance) and nearly a third were classed as NYHA III/IV.

Metrics of diastolic dysfunction (BNP, E/E’), and LV mass) were higher in HFpEF. Compared to control subjects (58 ± 5%), LVEF was marginally lower in HFpEF (56 ± 5%, p = 0.019), albeit preserved overall. More concentric remodeling (higher LV mass/volume) and diffuse fibrosis (iECV) was also evident in HFpEF. LA remodeling (higher LAVImax, LAVImin) and dysfunction (lower LAEF) was highly prevalent in HFpEF, irrespective of cardiac rhythm. RVEF in the control group had a narrow range (median 55, 47–70) in contrast to HFpEF (median 54, 27–73), but the difference was not statistically significant (p = 0.090). No significant differences in RV volumes between HFpEF and controls were noted.

### Comparison of HFpEF with and without RVD

RVD (was present in nearly one-fifth (19%, n = 25) of patients with HFpEF. Compared to patients without RVD, those with RVD presented more frequently with lower systolic blood pressure, AF, radiographic evidence of pulmonary congestion and elevated cardiothoracic ratio. RVD was associated with larger right ventricular and LA (LAVImin) volumes, and lower LAEF (irrespective of AF or sinus rhythm). Furthermore, prior hospitalization with decompensated HF was also more prevalent in this sub-group. There were no statistically significant differences between groups in terms of medical history, biochemical profiles and prescribed cardiac pharmacotherapies. While measures of functional status were worse in the RVD group i.e. greater proportion of NYHA III/IV and shorter 6 MWT distance walked, these differences did not reach statistical significance.

### Correlates of RVEF

Statistically significant correlations of RVEF with other variables are shown in Table 3. Positive correlations were observed with LVEF and LAEF. Inverse relationships were seen for RVEF with RV, LV and LA volumes. The strongest correlations were with RV volumes (right ventricular end-systolic volume indexed [RVESVI] r = − 0.788, right ventricular end-diastolic volume indexed [RVEDVI r = − 0.392] and LAEF r = 0.441).

**Table 3:** Significant associations of RVEF with other continuous variables

	Correlation coefficients (Spearman’s)	p value
Systolic BP (mmHg)	0.321	< 0.0001
LVEDVI (ml/m^2^)	0.174	0.044
LVEF (%)	0.219	0.011
RVEDVI (ml/m^2^)	− 0.392	< 0.0001
RVESVI (ml/m^2^)	− 0.788	< 0.0001
LAVImax (ml/m^2^)	− 0.175	0.043
LAVImin (ml/m^2^)	− 0.272	0.001
LAEF (%)	0.441	< 0.0001

Abbreviations are as per Tables 1 and 2

### RVD and outcomes

During median follow-up of 1429 days (1153–1654), 47% of HFpEF subjects (n = 64) experienced the composite endpoint of death (n = 22) or hospitalization with HF (n = 42). There were no events in the control group.

**Fig. 3:**
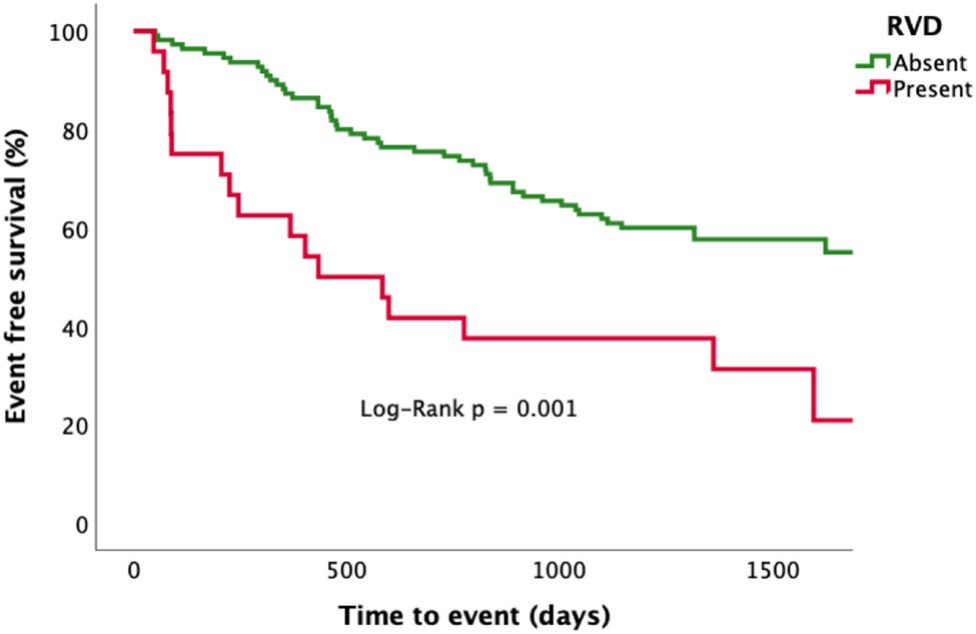
Survival analysis stratified according to the presence or absence of right ventricular dysfunction. Kaplan–Meier analysis for the composite endpoint of death and/or hospitalization with heart failure; *RVD* right ventricular dysfunction

Kaplan–Meier survival curves stratified according to the presence or absence of RVD in HFpEF are shown in Fig. 3. HFpEF patients with RVD had significantly higher event rates (Log-Rank p = 0.001). Furthermore, in those HFpEF patients with RVD, when stratified into tertiles on the basis of RVEF (see online Resource Supplementary Fig. 1), the lower RVEF groups were associated with increasing risk of HF hospitalizations, albeit statistical significance was not reached (Log-Rank p = 0.170). On univariable Cox regression analysis (Table 4), nineteen parameters were associated with adverse outcomes: age, diastolic blood pressure, prior HF hospitalization, NYHA III/IV, Log 6MWT distance, Log creatinine, haemoglobin, Log BNP, E/E’, left ventricular mass indexed (LVMI), LAVImax, LAEF, presence of MI on LGE, ECV indexed ECV, RVEDVI, RVESVI, Log RVEF and RVD. Of these, 4 parameters were excluded from multivariable analysis. During multivariable analysis (see Table 5), RVD remained significantly associated with outcomes in 3 separate models incorporating either: clinical, biochemical or imaging metrics. In a final model comprising the strongest parameters overall, RVD remained an independent predictor of outcomes (adjusted Hazard Ratio [HR] 3.946, 95% CI 1.878–8.290, p = 0.0001) along with indexed ECV (HR 1.742, CI 1.176–2.579, p = 0.006) and echocardiographic E/E’ (HR 1.745, CI 1.230–2.477, p = 0.002). The final Cox model incorporating these 3 independent variables to predict outcome yielded an area under the ROC curve of 0.732, p < 0.0001.

**Table 4:** Unadjusted predictors for the composite endpoint of death and/or hospitalization with heart failure

Unadjusted predictors of outcome		
	Hazard ratio (95%CI)	p value
Clinical		
Age (years)	1.406 (1.068–1.851)	0.015
Average diastolic BP (mmHg)	0.660 (0.505–0.863)	0.002
Prior HF hospitalization	3.332 (1.735–6.399)	0.0001
NYHA III/IV	1.747 (1.054–2.894)	0.030
Log 6MWT distance (m)	0.739 (0.580–0.941)	0.014
Clinical blood samples		
Log creatinine (μmol/l)	1.281 (1.021–1.607)	0.032
Haemoglobin (g/l)	0.711 (0.550–0.920)	0.009
Log BNP (ng/l)	1.437 (1.093–1.889)	0.009
Imaging		
LV mass index (g/m^2^)	1.284 (1.028–1.605)	0.028
LAVImax (ml/m^2^)	1.310 (1.044–1.643)	0.020
Biplane LAEF (%)	0.737 (0.578–0.938)	0.013
LGE MI (%)	1.745 (0.963–3.159)	0.066
RVEDVI (ml/m^2^)^a^	1.292 (1.001–1.668)	0.049
RVESVI (ml/m^2^)^a^	1.305 (1.035–1.645)	0.024
Log RVEF (%)^a^	0.819 (0.650–1.030)	0.088
RVD	2.533 (1.452–4.419)	0.001
ECV (%)^a^	1.578 (1.144–2.178)	0.005
iECV (ml/m^2^)	1.546 (1.128–2.119)	0.007
E/E′	1.420 (1.118–1.804)	0.004

^a^Parameters not entered into multivariable analysis; Abbreviations are as per Tables 1 and 2

**Table 5:** Multiple Cox regression models inclusive of RVD for the composite endpoint of death and/or hospitalization with heart failure

Adjusted predictors of outcome		
	Hazard ratio (95%CI)	p value
Clinical (Model 1)		
Age (years)	1.247 (0.925–1.679)	0.147
Average Diastolic BP (mmHg)	0.735 (0.572–0.944)	0.016
Prior HF hospitalization	2.671 (1.360–5.245)	0.004
NYHA III/IV	0.869 (0.391–1.929)	0.729
Log 6MWT distance (m)	0.883 (0.665–1.173)	0.392
+RVD	1.873 (1.054–3.327)	0.032
Clinical blood samples (model 2)		
Log Creatinine (μmol/l)	1.296 (1.034–1.624)	0.025
Haemoglobin (g/l)	0.764 (0.591–0.987)	0.040
Log BNP (ng/l)	1.228 (0.925–1.630)	0.155
+RVD	2.495 (1.419–4.384)	0.001
Imaging (model 3)		
LV mass index (g/m^2^)	0.848 (0.360–2.000)	0.707
LAVImax (ml/m^2^)	0.742 (0.414–1.332)	0.318
Biplane LAEF (%)	0.827 (0.575–1.189)	0.306
LGE MI (%)	1.374 (0.526–3.590)	0.516
iECV (ml/m^2^)	1.742 (1.176–2.579)	0.006
E/E′	1.745 (1.230–2.477)	0.002
+ RVD	3.946 (1.878–8.290)	0.0001
Strongest markers combined (model 4)		
Average diastolic BP (mmHg)	1.306 (0.890–1.916)	0.172
Prior HF hospitalization	2.094 (0.875–5.011)	0.097
Log creatinine (μmol/l)	1.343 (0.929–1.941)	0.116
Haemoglobin (g/l)	0.983 (0.634–1.525)	0.940
iECV (ml/m^2^)	1.742 (1.176–2.579)	0.006
E/E′	1.745 (1.230–2.477)	0.002
+ RVD	3.946 (1.878–8.290)	0.0001

Abbreviations are as per Tables 1 and 2

## Discussion

This is the first prospective study to analyze RV systolic performance and remodeling with CMR in both age- and sex-matched HFpEF and control groups. The principal findings in our study are that in HFpEF: (1) RVD is present in a significant minority; (2) RVEF is associated with RV/LV/LA volumes and LA function; and (3) RVD is independently associated with the risk of death or re-hospitalization with HF.

### Prevalence of RVD

To date, the reportedly wide range of prevalence of RVD in HFpEF of 4 to 44% has been derived almost exclusively from echocardiographic data [5]. Factors implicated in this variation in prevalence include the differing populations studied (community based, registry data, clinical trials) and variable definitions of both HFpEF (LVEF ≥ 45% and LVEF > 50%) and RVD [4, 5]. Besides, the complex geometry of the RV renders it a difficult chamber to assess with traditional 2D echocardiography, especially in the context of HFpEF when imaging may be more challenging due to co-morbidites such as lung disease, obesity and AF [4].

CMR is the established gold standard for RV assessment [6, 7]. To date, only one prior CMR study [8] has reported prevalence (19%) of RVD in HFpEF, using a RVEF cut-off of < 45%, primarily based upon ARVC guidelines [19]. In contrast, we observed a similar prevalence using a slightly higher RVEF cut-off of RVEF < 47% based on our own internal reference controls, a particular strength of our study.

### Significance of RVD in HFpEF, causes and possible mechanisms implicated in outcomes

In HFrEF, the presence of RVD portends poorer functional status, exercise capacity [1, 20] and prognosis [2]. However, a similar association of RVD with outcomes in HFpEF has not been observed consistently. In echocardiographic studies of community [21] and hospital based HFpEF subjects referred for invasive right heart catheterization [22], RVD was independently predictive of mortality. To the contrary, in a larger observational study [23] comprising outpatient HFpEF recruits and in the TOPCAT clinical trial [24], RVD did not adversely impact upon prognosis. The likely explanation for these differences include: variable HFpEF LVEF cut-offs, use of different parameters to define RVD as described earlier and more stringent exclusion criteria in clinical trials compared to community settings such as renal dysfunction or coronary artery disease which have been shown to be associated with RVD [4] but are also independently associated with increased risk [25].

Our work however adds to findings from the only 2 CMR-based HFpEF outcome studies to date [8, 9] and clearly implicates RVD as an important mediator of outcomes in HFpEF. In the first study [9], all surrogates of RVD, irrespective of modality (CMR, echocardiography and invasive right heart catheterization) were associated with death and or HF hospitalization during univariable analysis. In the above study (n = 142, median follow-up 10 months), a much lower CMR measured RVEF cut-off (< 35%) was used to define RVD in contrast to our study. However, this association with outcomes was not significant for CMR RVD during multivariable analysis, following adjustment for clinical variables. On the other hand echocardiographic (RV systolic function, estimated pulmonary artery systolic pressure [PASP]) and invasive (measured PASP, pulmonary capillary wedge pressure [PCWP]) metrics of RVD remained independent predictors of adverse outcomes. In the second study (n = 171, median follow-up 573 days), RVD measured by CMR outperformed echocardiographic-derived measures of RVD as a prognostic marker [8]. The RVEF cut-off to define RVD (< 45%) was also chosen based upon ROC analysis to detect end-points. In contrast to both of the aforementioned studies, our follow-up times were substantially longer, the mere presence of RVD and not just more severe RVD was significantly associated with worse outcomes in our cohort. Furthermore, our definition of RVD was based upon reference control data, again lacking in both of these prior studies.

In line with previous studies, the RVD sub-group in our HFpEF cohort was also noted to have lower systolic blood pressure, more frequent AF [26, 27], higher frequency of prior HF hospitalizations [21], a greater of adverse RV remodeling (RV enlargement) [27] and more prevalent pulmonary congestion [28]. There are likely multiple reasons for these findings which appear intimately linked. RV contractile function is intrinsically related to RV volumes, as also demonstrated by the moderate to strong inverse correlations of RVEF with RV volumes in our study. Furthermore, increasing RV size is an independent predictor of incident RVD development in HFpEF [27], analogous to that observed in similar LV pressure overloaded conditions such as aortic stenosis [29]. These factors either in isolation or when coupled together are known to be associated with increased venous congestion [28] as also shown by the higher rates of congestive chest radiographic changes in our RVD subjects. Increasing congestion is a major cause of HF hospitalization and therefore likely explains the observation of both prior HF hospitalization as well the association with re-hospitalization with HF (as a part of the composite end-point) seen in our RVD sub-group. Both the RVD subjects from our cohort and from previous studies [8, 9] also demonstrate an association with increased LA size, a surrogate marker of high LA pressure, which likely portends congestion. Furthermore, the RVD group also had a greater proportion of AF, which is known to further exacerbate RV contractile dysfunction [22, 26, 27] and provoke circulatory collapse [20] likely necessitating HF hospitalizations [30]. Our study is also the first to demonstrate an association between CMR RVEF and LAEF which likely further compounds the above features and has been hypothesized previously from echocardiographic data [22].

Other authors have previously suggested a clear relationship between RVD and the severity of left heart disease as reflected by NYHA class, natriuretic peptides or LV systolic function [5]. However, in our study, these parameters were not different between those with and without RVD. This may merely be a reflection of our sample size. Alternatively, RVD may be part of the aetiological profile in HFpEF whereby biventricular remodeling often co-exists, even in early stages [31] or driven by diffuse fibrosis secondary to systemic inflammation affecting both ventricles [32]. The correlations, albeit weak observed between RVEF and LVEF/left ventricular end-diastolic volume indexed (LVEDVI) in our study may also be explained by a degree of ventricular interdependence driven by obesity, typical of HFpEF populations [27].

Although the observational nature of our study precludes determination of causation, AF was significantly associated with RVD, suggesting a contributory role. Our findings of a higher AF prevalence are consistent with similar reports from previous HFpEF studies [8, 21, 22, 24]. However, it remains unclear whether AF is a cause or consequence of RVD in HF [20]. In HFrEF, RVD reportedly predicts future AF development [33]. Irrespective of HF subtype or aetiology, AF in the setting of RVD is associated with haemodynamic instability and with poorer outcomes [20, 34]. In the HFpEF population at large, development of AF confers a poorer quality of life [30], increases hospitalization rates and worsens mortality [30, 35].

### Potential implications of our study

Our results, through the gold standard medium of CMR reinforce previous data that RVD is present in a significant minority of HFpEF. Furthermore, the presence of RVD alone and not necessarily more severe RVD is associated with heightened risk in HFpEF. RVD may drive recurrent HF hospitalizations and mortality. Identifying RVD is potentially important for multiple reasons. HF hospitalizations are associated with significant morbidity and are a drain on healthcare resources [36]. Importantly, the prevalence of HFpEF is rising [3]. Understanding the mechanistic triggers for decompensation in HFpEF may also enable targeted therapies (e.g. RV focused, management of AF). Whilst treatments in unselected HFpEF patients have been neutral at best [37], one small study addressing pulmonary hypertension and RVD using a phosphodiesterase-5 inhibitor showed significant improvements in both cardiac haemodynamics and RV function [38].

### Strengths and limitations

Our study is one of the largest to date evaluating RV performance utilizing CMR and also benefits from having the longest follow-up to gauge the impact of RVD on clinical outcomes. Furthermore, we also have a comparator control group which is a particular strength. While RVEF measurement is reportedly more reproducible using axial slice orientations [39], we deliberately assessed RV function from the short axis orientation since this is the method used routinely in clinical practice and our normative data were also derived using the same methodology [16]. Importantly, our technique yielded excellent intra- and inter-observer agreements.

This is a single centre, observational study and therefore should be replicated in additional cohorts. The association between RVD and outcome does not imply causality. We also do not have outcome data for deaths categorized as cardiovascular versus non-cardiovascular. Our definition of HFpEF was not in accordance with latest European Society of Cardiology guidelines [40]. In particular diastolic dysfunction nor elevated natriuretic peptide levels were required for diagnosis. However, diastolic dysfunction at rest is reportedly absent in nearly one-third of contemporary HFpEF clinical trials [41] and conversely also identified in a significant proportion of asymptomatic elderly subjects [41]. Only a small minority of HFpEF patients in our study had BNP levels below ESC diagnostic thresholds (14%) which is unsurprising given the high burden of obesity observed [42]. During screening, all of our HFpEF patients subsequently enrolled had already had a diagnostic label of HFpEF made by Consultant Cardiologists during prior outpatient clinics or following a HF hospitalization. Our control group included some hypertensive subjects and was therefore not totally devoid of cardiovascular disease. Since we excluded severe lung disease (which can cause RVD), our reported prevalence of RVD is probably lower than in the general HFpEF population at large. We did not calculate (derive) estimates of pulmonary artery pressures (PAP) or quantify tricuspid regurgitation severity using echocardiography or directly assess PAP using right heart catheterization.

## Conclusions

RVD as assessed by CMR is present in a significant proportion of HFpEF and is independently associated with death and or HF hospitalizations.

## Electronic supplementary material

Below is the link to the electronic supplementary material.

Supplementary file1 (PDF 383 kb)
