# Supplementary material for: Prevalence of right ventricular dysfunction and prognostic significance in heart failure with preserved ejection fraction
Source: Int J Cardiovasc Imaging. 2020 Jul 31;37(1):255–66. doi: 10.1007/s10554-020-01953-y (PMC7878207; doi:10.1007/s10554-020-01953-y)
Supplement: Supplementary file 1 — Supplementary file1 (PDF 383 kb) [file 10554_2020_1953_MOESM1_ESM.pdf]

Title

Prevalence of right ventricular dysfunction and prognostic significance in heart failure with preserved ejection fraction

Journal

The International Journal of Cardiovascular Imaging

Names of authors and affiliations

Prathap Kanagala[a, b] – MBBS, PhD pkk12@leicester.ac.uk

Jayanth R. Arnold[a] – BMBChB, DPhil jra14@leicester.ac.uk

Anvesha Singh[a] – MBChB, PhD as707@leicester.ac.uk

Jamal N. Khan[a] – MBChB, PhD mally777@hotmail.com

Gaurav S. Gulsin[a] – gg149@leicester.ac.uk

Pankaj Gupta[a] – DPB, FRCPath pankaj_gupta54@hotmail.com

Iain B. Squire[a] – MBChB, MD is11@leicester.ac.uk

Leong L. Ng[a] – MB B Chir, MD lln1@leicester.ac.uk

Gerry P. McCann[a] – MBChB, MD gpm12@leicester.ac.uk

From the Department of Cardiovascular Sciences, University of Leicester, National Institute for Health Research (NIHR) Leicester Biomedical Research Centre, Leicester, United Kingdom[a] and Aintree University Hospital, Liverpool, United Kingdom[b]

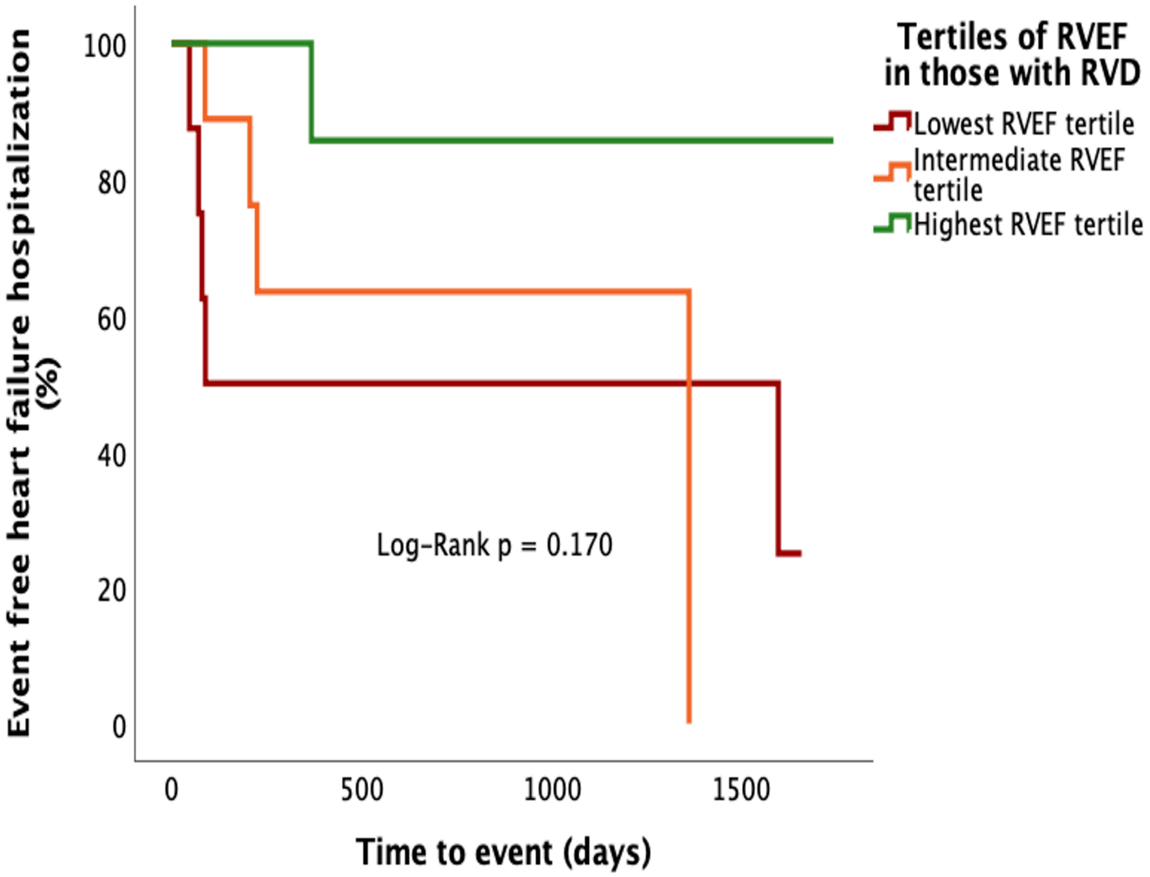

Online Resource Supplementary Fig. 1 Association of worsening right ventricular ejection fraction with risk of heart failure hospitalization in those with right ventricular dysfunction and heart failure with preserved ejection fraction

Caption: Kaplan-Meier analysis for the endpoint of hospitalization with heart failure in those with RVD, stratified according to tertiles of RVEF; RVD = right ventricular dysfunction; RVEF = right ventricular ejection fraction
